# 4-{[4-(Hy­droxy­meth­yl)piperidin-1-yl]meth­yl}phenol

**DOI:** 10.1107/S1600536812028838

**Published:** 2012-06-30

**Authors:** M. C. R. Simões, I. M. R. Landre, M. S. Moreira, C. Viegas Jr, A. C. Doriguetto

**Affiliations:** a Laboratório de Fitoquímica e Química Medicinal, Instituto de Química, Universidade Federal de Alfenas (UNIFAL–MG), Alfenas, MG, Brazil; b Laboratório de Cristalografia, Instituto de Química, Universidade Federal de Alfenas (UNIFAL–MG), Alfenas, MG, Brazil

## Abstract

In the title compound, C_13_H_19_NO_2_, the piperidine ring has a chair conformation with the exocyclic N—C bond in an equatorial position. In the crystal, mol­ecules are linked head-to-tail by phenol O—H⋯O hydrogen bonds to hy­droxy­methyl­ene O-atom acceptors, forming chains which extend along [100]. These chains form two-dimensional networks lying parallel to (101) through cyclic hydrogen-bonding associations [graph set *R*_4_^4^(30)], involving hy­droxy O—H donors and piperidine N-atom acceptors.

## Related literature

For preparative procedures of the title compound and related compounds, see: Kulagowski *et al.* (1996 ▶); Schepartz & Breslow (1987) ▶; Menegatti *et al.* (2003 ▶). For physiological properties of these compounds, see: Menegatti *et al.* (2003 ▶); Romero *et al.* (2003 ▶). For ring conformations, see: Domenicano *et al.* (1975 ▶). For graph-set analysis, see: Etter *et al.* (1990 ▶).
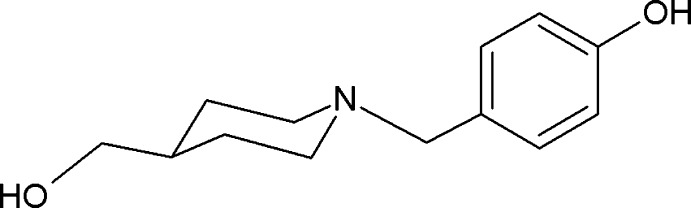


## Experimental

### 

#### Crystal data

C_13_H_19_NO_2_
*M* *_r_* = 221.29
Monoclinic, 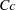

*a* = 6.0428 (2) Å
*b* = 17.2269 (7) Å
*c* = 11.3010 (4) Å
β = 94.663 (4)°
*V* = 1172.53 (7) Å^3^
*Z* = 4
Mo *K*α radiation
μ = 0.08 mm^−1^
*T* = 150 K
0.64 × 0.15 × 0.07 mm

#### Data collection

Oxford Diffraction Xcalibur Atlas Gemini Ultra CCD diffractometer
5289 measured reflections
1474 independent reflections
1326 reflections with *I* > 2σ(*I*)
*R*_int_ = 0.021

#### Refinement

*R*[*F* ^2^ > 2σ(*F* ^2^)] = 0.033
*wR*(*F* ^2^) = 0.084
*S* = 1.08
1474 reflections
151 parameters
2 restraints
H atoms treated by a mixture of independent and constrained refinement
Δρ_max_ = 0.23 e Å^−3^
Δρ_min_ = −0.16 e Å^−3^

### 

Data collection: *CrysAlis PRO* (Oxford Diffraction, 2010 ▶); cell refinement: *CrysAlis PRO*; data reduction: *CrysAlis PRO*; program(s) used to solve structure: *SIR2004* (Burla *et al.*, 2005 ▶); program(s) used to refine structure: *SHELXL97* (Sheldrick, 2008 ▶); molecular graphics: *ORTEP-3 for Windows* (Farrugia, 1997 ▶) and *Mercury* (Macrae *et al.*, 2006 ▶); software used to prepare material for publication: *WinGX* (Farrugia, 1999 ▶).

## Supplementary Material

Crystal structure: contains datablock(s) global, I. DOI: 10.1107/S1600536812028838/zs2215sup1.cif

Structure factors: contains datablock(s) I. DOI: 10.1107/S1600536812028838/zs2215Isup2.hkl

Supplementary material file. DOI: 10.1107/S1600536812028838/zs2215Isup3.cml

Additional supplementary materials:  crystallographic information; 3D view; checkCIF report

## Figures and Tables

**Table 1:** Hydrogen-bond geometry (Å, °)

*D*—H⋯*A*	*D*—H	H⋯*A*	*D*⋯*A*	*D*—H⋯*A*
O1—H1⋯N1^i^	0.78 (3)	2.04 (3)	2.813 (2)	174 (3)
O2—H2⋯O1^ii^	0.89 (2)	1.82 (2)	2.702 (2)	176 (2)

Symmetry codes: (i) 
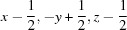
; (ii) 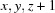.

**Fig. 1.**
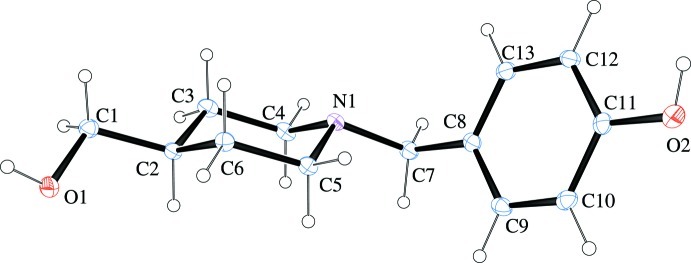
Molecular conformation and atom-numbering scheme for the title compound. Displacement ellipsoids for non-H atoms are drawn at the 30% probability level.

**Fig. 2.**
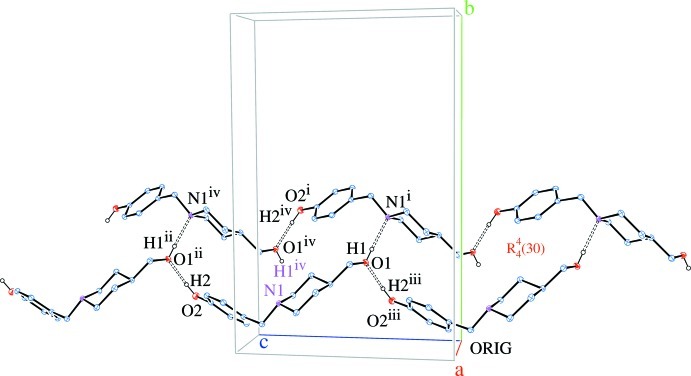
The crystal packing of (I) showing the chains formed along [001]. Hydrogen bonds are shown as dashed lines. [For symmetry codes (i) and (ii), see Table 1. For other codes: (iii) x, y, z - 1; (iv) x - 1/2, -y + 1/2, z + 1/2; (v) x - 1/2, -y + 1/2, z - 1/2].

**Fig. 3.**
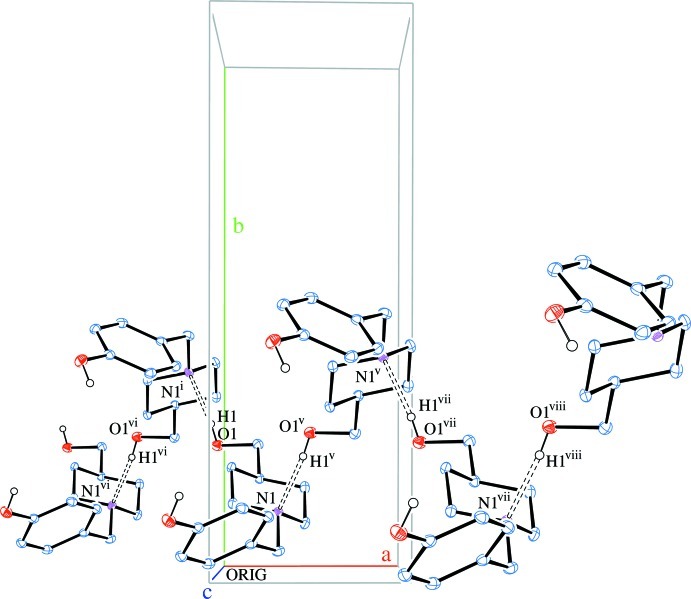
The crystal packing showing the chain extension formed along [101]. Hydrogen bonds are shown as dashed lines. [For symmetry codes (i) and (v), see Table 1 and Fig. 1. For other codes: (vi) x - 1, y, z - 1; (vii) x + 1, y, z + 1; (viii) x + 3/2, -y + 1/2, z + 3/2].

## supplementary crystallographic information

### Comment

A wide range of benzylpiperazine and benzylpiperidine derivatives are reported in the literature as products or intermediates in the synthesis of new compound prototypes (Kulagowski *et al.*, 1996) showing various biological activities, including antipsychotic (Menegatti *et al.*, 2003) and antidepressant properties (Romero *et al.*, 2003). These compounds can be obtained by the reaction of aldehydes or ketones with primary and secondary amines using a reducing agent. In this context, the title compound 4-((4-(hydroxymethyl)piperidin-1-yl)methyl)phenol, C_13_H_19_NO_2_ was prepared from 4-hydroxybenzaldehyde and 4-piperidinemethanol (Menegatti *et al.*, 2003; Schepartz & Breslow, 1987) with a 96% yield. In this compound (Fig. 1) the mean plane through the C7 and phenolic atoms shows that this moiety is, as expected, planar (r.m.s deviation = 0.0121 Å). Considering the non-H atoms, the largest deviation from the least-squares plane is 0.021 (1) Å for C7. The H2 atom deviates -0.23 (2) Å from the least-squares plane due to its involvement in intermolecular H bonds. The least-squares planes through atoms C3—C4—N1—C7—C8 [r.m.s = 0.0149 Å and largest deviation = 0.019 (1) Å for C4] and C6—C5—N1—C7 [r.m.s. =0.0233 Å and largest deviation = 0.0243 (8) Å for C5], show that these moieties are also individually very planar and form dihedral angles of 77.38 (9) and 76.65 (7)° with the phenolic ring. The piperidine ring has a chair conformation with a weighted average absolute torsion angle of 56.85(6,52)° (1st is the e.s.d. internal and 2nd is the external one) (Domenicano *et al.*, 1975). Considering the two possible chair conformations of piperidine rings, the N1—C7 bond is an equatorial orientation.

There are two independent classic hydrogen-bond types involving the phenolic, hydroxymethylene and piperidine groups, contributing to the stability of the crystal packing (Table 1). Translation-related molecules are linked head-to-tail along [001] through phenolic O—H···O hydrogen bonds to hydroxymethylene O-atom acceptors (Fig. 2). This hydroxy group also acts as a donor to the piperidine nitrogen (Fig. 3), forming two-dimensional sheets which extend across (010). The morphology of the cyclic H-bond pattern within the sheets is *R*_4_^4^(30) (Etter *et al.*, 1990). No π-π interactions are present in the structure.

### Experimental

4-Hydroxybenzaldehyde (0.3 g) and 4-piperidinemethanol (0.27 g) were dissolved in 8.5 mL of methanol. The pH was adjusted to 5 with the addition of acetic acid before the addition of 0.15 g of sodium cyanoborohydride. The system was kept stirred under reflux for 5 h, followed by addition of concentrated HCl (ca. 2 mL) to pH 2.0 and the resulting solution was then basified to pH 12 with solid NaOH. The reaction mixture was extracted with chloroform (3 *x* 15 mL), and the combined organic phase was subsequently washed with water, then brine, dried over anhydrous sodium sulfate, followed by filtration. However, it was observed that the product was present mainly in the aqueous layer and after a slow evaporation of the solvent, crystalline material (m.p. 329 K) suitable for X-ray diffraction formed. IR (KBr, cm^-1^): ν 3444, 2334, 1573, 1413, and 1012.

### Refinement

Positional and anisotropic displacement parameters were refined for all non-H atoms. The H atoms of the aromatic and aliphatic groups were positioned stereochemically and were refined with fixed individual displacement parameters [*U*_iso_(H) = 1.2*U*_eq_(C)] using a riding model with C—H_(aromatic)_ = 0.95 Å and C—H_(aliphatic)_ = 0.99 Å. The hydroxyl H atoms were located by difference-Fourier synthesis and were set as isotropic [*U*_iso_(H) = 1.5*U*_eq_(O)]. In the absence of significant anomalous scattering, the Friedel pair reflections were merged before the final refinement.

### Crystal data

**Table:**

C_13_H_19_NO_2_	*F*(000) = 480
*M**_r_* = 221.29	*D*_x_ = 1.254 Mg m^−^^3^
Monoclinic, *C**c*	Melting point: 329 K
Hall symbol: C -2yc	Mo *K*α radiation, λ = 0.71073 Å
*a* = 6.0428 (2) Å	Cell parameters from 3165 reflections
*b* = 17.2269 (7) Å	θ = 3.0–29.4°
*c* = 11.3010 (4) Å	µ = 0.08 mm^−^^1^
β = 94.663 (4)°	*T* = 150 K
*V* = 1172.53 (7) Å^3^	Prism, colourless
*Z* = 4	0.64 × 0.15 × 0.07 mm

### Data collection

**Table:**

Oxford Diffraction Xcalibur Atlas Gemini Ultra CCD diffractometer	1326 reflections with *I* > 2σ(*I*)
Radiation source: Enhance (Mo) X-ray Source	*R*_int_ = 0.021
Graphite monochromator	θ_max_ = 29.5°, θ_min_ = 3.0°
Detector resolution: 10.4186 pixels mm^-1^	*h* = −8→7
ω scans	*k* = −23→21
5289 measured reflections	*l* = −14→15
1474 independent reflections	

### Refinement

**Table:**

Refinement on *F*^2^	2 restraints
Least-squares matrix: full	H atoms treated by a mixture of independent and constrained refinement
*R*[*F*^2^ > 2σ(*F*^2^)] = 0.033	*w* = 1/[σ^2^(*F*_o_^2^) + (0.0533*P*)^2^] where *P* = (*F*_o_^2^ + 2*F*_c_^2^)/3
*wR*(*F*^2^) = 0.084	(Δ/σ)_max_ < 0.001
*S* = 1.08	Δρ_max_ = 0.23 e Å^−^^3^
1474 reflections	Δρ_min_ = −0.16 e Å^−^^3^
151 parameters	

### Special details

**Table:**

Geometry. All s.u.'s (except the s.u. in the dihedral angle between two l.s. planes) are estimated using the full covariance matrix. The cell s.u.'s are taken into account individually in the estimation of s.u.'s in distances, angles and torsion angles; correlations between s.u.'s in cell parameters are only used when they are defined by crystal symmetry. An approximate (isotropic) treatment of cell s.u.'s is used for estimating s.u.'s involving l.s. planes.
Refinement. Refinement of *F*^2^ against ALL reflections. The weighted *R*-factor *wR* and goodness of fit *S* are based on *F*^2^, conventional *R*-factors *R* are based on *F*, with *F* set to zero for negative *F*^2^. The threshold expression of *F*^2^ > σ(*F*^2^) is used only for calculating *R*-factors(gt) *etc*. and is not relevant to the choice of reflections for refinement. *R*-factors based on *F*^2^ are statistically about twice as large as those based on *F*, and *R*- factors based on ALL data will be even larger.

### Fractional atomic coordinates and isotropic or equivalent isotropic displacement parameters (Å^2^)

**Table:**

	*x*	*y*	*z*	*U*_iso_*/*U*_eq_	
O1	−0.0007 (2)	0.24016 (8)	0.46384 (12)	0.0261 (3)	
O2	−0.1558 (2)	0.11336 (9)	1.34326 (13)	0.0288 (3)	
N1	0.3345 (2)	0.12022 (8)	0.86219 (13)	0.0188 (3)	
C7	0.3394 (3)	0.05377 (11)	0.94654 (16)	0.0240 (4)	
H7A	0.4956	0.0424	0.9743	0.029*	
H7B	0.2779	0.0073	0.9041	0.029*	
C2	0.2291 (3)	0.17910 (11)	0.61963 (15)	0.0194 (4)	
H2A	0.1677	0.1302	0.5823	0.023*	
C1	0.2165 (3)	0.24167 (10)	0.52460 (17)	0.0230 (4)	
H1A	0.2455	0.2932	0.5614	0.028*	
H1B	0.3299	0.232	0.4678	0.028*	
C9	0.0023 (3)	0.03522 (10)	1.06266 (17)	0.0245 (4)	
H9	−0.0584	0.0015	1.0019	0.029*	
C8	0.2102 (3)	0.06858 (10)	1.05249 (15)	0.0216 (4)	
C6	0.0905 (3)	0.19913 (10)	0.72254 (16)	0.0200 (4)	
H6A	0.1448	0.2484	0.7596	0.024*	
H6B	−0.0662	0.2067	0.692	0.024*	
C12	0.1777 (3)	0.13204 (10)	1.24159 (16)	0.0232 (4)	
H12	0.24	0.1648	1.3032	0.028*	
C10	−0.1178 (3)	0.05027 (11)	1.15973 (17)	0.0245 (4)	
H10	−0.2592	0.0269	1.1648	0.029*	
C5	0.1038 (3)	0.13527 (10)	0.81530 (15)	0.0201 (4)	
H5A	0.015	0.1504	0.8815	0.024*	
H5B	0.0391	0.0871	0.7796	0.024*	
C13	0.2946 (3)	0.11669 (10)	1.14421 (16)	0.0235 (4)	
H13	0.4366	0.1396	1.1397	0.028*	
C3	0.4675 (3)	0.16258 (11)	0.66953 (17)	0.0239 (4)	
H3A	0.5569	0.1457	0.6046	0.029*	
H3B	0.5348	0.2106	0.7045	0.029*	
C11	−0.0317 (3)	0.09943 (10)	1.24953 (16)	0.0221 (4)	
C4	0.4701 (3)	0.09956 (11)	0.76403 (16)	0.0227 (4)	
H4A	0.4131	0.0506	0.727	0.027*	
H4B	0.6251	0.0904	0.7963	0.027*	
H1	−0.038 (4)	0.2801 (15)	0.437 (2)	0.034*	
H2	−0.106 (4)	0.1540 (14)	1.386 (2)	0.034*	

### Atomic displacement parameters (Å^2^)

**Table:**

	*U*^11^	*U*^22^	*U*^33^	*U*^12^	*U*^13^	*U*^23^
O1	0.0303 (7)	0.0222 (6)	0.0243 (7)	0.0004 (6)	−0.0057 (6)	0.0034 (5)
O2	0.0317 (7)	0.0280 (7)	0.0271 (7)	−0.0054 (6)	0.0058 (6)	0.0010 (6)
N1	0.0180 (7)	0.0199 (7)	0.0180 (7)	0.0020 (6)	−0.0014 (6)	−0.0029 (5)
C7	0.0284 (10)	0.0193 (8)	0.0234 (9)	0.0037 (7)	−0.0040 (8)	−0.0005 (7)
C2	0.0192 (9)	0.0208 (8)	0.0181 (8)	−0.0012 (7)	0.0008 (7)	−0.0018 (7)
C1	0.0231 (9)	0.0238 (9)	0.0223 (9)	−0.0021 (7)	0.0027 (7)	−0.0015 (7)
C9	0.0294 (10)	0.0190 (8)	0.0236 (9)	0.0004 (8)	−0.0070 (8)	0.0002 (7)
C8	0.0259 (9)	0.0174 (8)	0.0207 (9)	0.0032 (7)	−0.0029 (7)	0.0020 (6)
C6	0.0154 (8)	0.0216 (8)	0.0227 (8)	0.0028 (7)	0.0003 (7)	0.0004 (7)
C12	0.0269 (9)	0.0198 (8)	0.0218 (9)	−0.0028 (7)	−0.0038 (7)	−0.0014 (7)
C10	0.0241 (9)	0.0203 (9)	0.0281 (10)	−0.0023 (7)	−0.0034 (8)	0.0061 (7)
C5	0.0166 (8)	0.0228 (9)	0.0208 (9)	0.0017 (7)	0.0007 (7)	−0.0013 (7)
C13	0.0234 (10)	0.0215 (9)	0.0251 (9)	−0.0032 (7)	−0.0017 (7)	0.0020 (7)
C3	0.0181 (9)	0.0299 (10)	0.0239 (9)	−0.0001 (7)	0.0026 (7)	−0.0046 (8)
C11	0.0253 (9)	0.0196 (8)	0.0209 (9)	0.0021 (8)	−0.0004 (7)	0.0058 (7)
C4	0.0181 (8)	0.0273 (9)	0.0223 (9)	0.0053 (8)	−0.0003 (7)	−0.0058 (7)

### Geometric parameters (Å, º)

**Table:**

O1—C1	1.431 (2)	C9—H9	0.95
O1—H1	0.78 (3)	C8—C13	1.391 (2)
O2—C11	1.368 (2)	C6—C5	1.517 (2)
O2—H2	0.89 (2)	C6—H6A	0.99
N1—C5	1.474 (2)	C6—H6B	0.99
N1—C4	1.475 (2)	C12—C13	1.380 (3)
N1—C7	1.488 (2)	C12—C11	1.394 (3)
C7—C8	1.503 (3)	C12—H12	0.95
C7—H7A	0.99	C10—C11	1.390 (3)
C7—H7B	0.99	C10—H10	0.95
C2—C1	1.519 (3)	C5—H5A	0.99
C2—C6	1.527 (2)	C5—H5B	0.99
C2—C3	1.531 (2)	C13—H13	0.95
C2—H2A	1	C3—C4	1.522 (3)
C1—H1A	0.99	C3—H3A	0.99
C1—H1B	0.99	C3—H3B	0.99
C9—C10	1.388 (3)	C4—H4A	0.99
C9—C8	1.395 (3)	C4—H4B	0.99
			
C1—O1—H1	113.4 (19)	C2—C6—H6B	109.4
C11—O2—H2	111.9 (16)	H6A—C6—H6B	108
C5—N1—C4	109.81 (13)	C13—C12—C11	120.00 (17)
C5—N1—C7	109.54 (14)	C13—C12—H12	120
C4—N1—C7	108.23 (13)	C11—C12—H12	120
N1—C7—C8	113.27 (14)	C9—C10—C11	120.13 (17)
N1—C7—H7A	108.9	C9—C10—H10	119.9
C8—C7—H7A	108.9	C11—C10—H10	119.9
N1—C7—H7B	108.9	N1—C5—C6	111.80 (14)
C8—C7—H7B	108.9	N1—C5—H5A	109.3
H7A—C7—H7B	107.7	C6—C5—H5A	109.3
C1—C2—C6	112.29 (15)	N1—C5—H5B	109.3
C1—C2—C3	112.57 (15)	C6—C5—H5B	109.3
C6—C2—C3	108.63 (13)	H5A—C5—H5B	107.9
C1—C2—H2A	107.7	C12—C13—C8	121.87 (17)
C6—C2—H2A	107.7	C12—C13—H13	119.1
C3—C2—H2A	107.7	C8—C13—H13	119.1
O1—C1—C2	108.50 (14)	C4—C3—C2	110.28 (15)
O1—C1—H1A	110	C4—C3—H3A	109.6
C2—C1—H1A	110	C2—C3—H3A	109.6
O1—C1—H1B	110	C4—C3—H3B	109.6
C2—C1—H1B	110	C2—C3—H3B	109.6
H1A—C1—H1B	108.4	H3A—C3—H3B	108.1
C10—C9—C8	121.37 (16)	O2—C11—C10	118.44 (17)
C10—C9—H9	119.3	O2—C11—C12	122.46 (17)
C8—C9—H9	119.3	C10—C11—C12	119.10 (17)
C13—C8—C9	117.51 (17)	N1—C4—C3	112.40 (14)
C13—C8—C7	120.81 (16)	N1—C4—H4A	109.1
C9—C8—C7	121.68 (16)	C3—C4—H4A	109.1
C5—C6—C2	111.16 (14)	N1—C4—H4B	109.1
C5—C6—H6A	109.4	C3—C4—H4B	109.1
C2—C6—H6A	109.4	H4A—C4—H4B	107.9
C5—C6—H6B	109.4		
			
C5—N1—C7—C8	−61.51 (17)	C2—C6—C5—N1	57.80 (18)
C4—N1—C7—C8	178.78 (14)	C11—C12—C13—C8	−0.5 (3)
C6—C2—C1—O1	−70.76 (18)	C9—C8—C13—C12	−0.7 (3)
C3—C2—C1—O1	166.27 (13)	C7—C8—C13—C12	179.08 (16)
C10—C9—C8—C13	0.9 (3)	C1—C2—C3—C4	−179.95 (16)
C10—C9—C8—C7	−178.82 (16)	C6—C2—C3—C4	55.06 (18)
N1—C7—C8—C13	−75.6 (2)	C9—C10—C11—O2	179.45 (15)
N1—C7—C8—C9	104.14 (19)	C9—C10—C11—C12	−1.1 (2)
C1—C2—C6—C5	179.30 (15)	C13—C12—C11—O2	−179.23 (16)
C3—C2—C6—C5	−55.54 (18)	C13—C12—C11—C10	1.4 (2)
C8—C9—C10—C11	0.0 (3)	C5—N1—C4—C3	57.90 (18)
C4—N1—C5—C6	−57.51 (17)	C7—N1—C4—C3	177.44 (15)
C7—N1—C5—C6	−176.24 (14)	C2—C3—C4—N1	−57.70 (19)

### Hydrogen-bond geometry (Å, º)

**Table:**

*D*—H···*A*	*D*—H	H···*A*	*D*···*A*	*D*—H···*A*
O1—H1···N1^i^	0.78 (3)	2.04 (3)	2.813 (2)	174 (3)
O2—H2···O1^ii^	0.89 (2)	1.82 (2)	2.702 (2)	176 (2)

Symmetry codes: (i) *x*−1/2, −*y*+1/2, *z*−1/2; (ii) *x*, *y*, *z*+1.
